# Anode potential influences the structure and function of anodic electrode and electrolyte-associated microbiomes

**DOI:** 10.1038/srep39114

**Published:** 2016-12-19

**Authors:** Paul G. Dennis, Bernardino Virdis, Inka Vanwonterghem, Alif Hassan, Phil Hugenholtz, Gene W. Tyson, Korneel Rabaey

**Affiliations:** 1 School of Agriculture and Food Sciences, The University of Queensland, Brisbane, Queensland 4072, Australia; 2 Advanced Water Management Centre, The University of Queensland, Brisbane, Queensland 4072, Australia; 3 Australian Centre for Ecogenomics, The University of Queensland, Brisbane, Queensland 4072, Australia; 4 Centre for Microbial Electrochemical Systems, The University of Queensland, Brisbane, Queensland 4072, Australia; 5 Laboratory of Microbial Ecology and Technology, Ghent University, Coupure Links 653 9000 Ghent, Belgium

## Abstract

Three bioelectrochemical systems were operated with set anode potentials of +300 mV, +550 mV and +800 mV vs. Standard Hydrogen Electrode (SHE) to test the hypothesis that anode potential influences microbial diversity and is positively associated with microbial biomass and activity. Bacterial and archaeal diversity was characterized using 16 S rRNA gene amplicon sequencing, and biofilm thickness was measured as a proxy for biomass. Current production and substrate utilization patterns were used as measures of microbial activity and the mid-point potentials of putative terminal oxidases were assessed using cyclic voltammetry. All measurements were performed after 4, 16, 23, 30 and 38 days. Microbial biomass and activity differed significantly between anode potentials and were lower at the highest potential. Anodic electrode and electrolyte associated community composition was also significantly influenced by anode potential. While biofilms at +800 mV were thinner, transferred less charge and oxidized less substrate than those at lower potentials, they were also associated with putative terminal oxidases with higher mid-point potentials and generated more biomass per unit charge. This indicates that microbes at +800 mV were unable to capitalize on the potential for additional energy gain due to a lack of adaptive traits to high potential solid electron acceptors and/or sensitivity to oxidative stress.

Bioelectrochemical systems (BESs) use microorganisms to catalyse chemical reactions at electrodes and have an increasingly diverse portfolio of applications^1^. The most commonly described BES is the microbial fuel cell (MFC), in which electrical power is generated via microbial decomposition of organic matter at an anode coupled with cathodic reduction of an electron acceptor at more positive potential. This process is facilitated by terminal reductases such as redox-active proteins, soluble mediators and/or conductive structures (e.g. “nanowires”) that allow extracellular transfer of electrons from microbial respiratory chains to solid electron acceptors such as anodic electrodes^2^.

Thermodynamics dictates that microorganisms should obtain more energy when using anodes at higher electrochemical potentials, as long as they can use the energy to generate additional proton motive force^3^. If this is the case, anode potential should be positively associated with current generation and microbial biomass, and influence microbial diversity; however, the relationship between anode potential and microbial community structure and function is not clear^4^.

Positive associations between anode potential (reported as values relative to the Standard Hydrogen Electrode (SHE) throughout this paper) and current have been reported in the approximate ranges −200 mV to +100 mV^5^, +100 mV and +600 mV^6^ and +250 to +800 mV^7^. However, Torres *et al*.^8^ detected a negative association between anode potential and current in the range −150 mV to +370 mV. In addition, other studies indicate unimodal relationships, in which current remains similar at approximately 300 mV, 700 mV and 900 mV but drops significantly at 600 mV^9^ or peaks between approximately −50 mV to +400 mV and declines sharply outside of this range^4^,^10^.

Unimodal relationships have also been observed between anode potential and biomass, where biomass peaks at 0 mV to 400 mV^10^ or 200 mV to 500 mV^4^ and then declines at more positive or negative potentials. Consistent with these findings, Aelterman *et al*.^11^ found that biomass was similar between 0 mV and −200 mV and then declined slightly between −200 mV and −400 mV. In other studies, however, the relationship between anode potential and biomass cannot be assessed as biomass measurements are typically neglected.

Most studies indicate that anode potential influences the composition of anodic-electrode-associated microbial communities, i.e. different anode potentials select for different populations^5^,^8^,^12^. Few studies, however, consider the influence of anode potential on the number of taxa and/or the evenness of population abundances. An exception to this is the study of Torres *et al*.^8^ in which a positive correlation was observed between the number of anodic-electrode-associated microbial taxa and anode potential. In contrast, Zhu *et al*.^4^ observed no effects of anode potential on microbial diversity between −250 mV to 810 mV, despite significant changes in reactor performance. Based on these results, Zhu *et al*.^4^ concluded that physiological changes in the organisms present, rather than shifts in community structure, explain the association between anode potential and reactor performance. However, all studies to date that concern the influence of anode potential on microbial diversity are based on single or duplicate measurements at one or two time points. Consequently, the conclusions drawn from these studies lack appropriate statistical support and the temporal dynamics of microbial communities exposed to different anode potentials are poorly characterized. Lastly, with few exceptions^5^, most studies do not characterize anodic-electrolyte-associated communities, so it is not known whether they respond to anode potential.

In this study, we tested the hypothesis that anode potential influences microbial diversity and is positively associated with microbial biomass and activity. To achieve this, three BESs were inoculated with a diverse microbial community and operated at different anode potentials: +300, +550 and +800 mV *vs* SHE (Figs S1 and S2), while receiving a continuous feed of mixed volatile fatty acids (VFAs). Each reactor featured 15 exchangeable anodes that enabled biofilms to be characterized in triplicate at five time points^13^. The diversity of anodic electrode and electrolyte associated communities was characterized using 16 S rRNA gene amplicon sequencing and anodic electrode associated microbial biomass was determined by measuring biofilm thickness. Microbial catalytic activity was assessed by monitoring current generation and substrate utilisation patterns over time. Finally, cyclic voltammetry was used to determine the mid-point potential of the putative terminal oxidases associated with anodic biofilms in each reactor.

## Results

### Current generation and substrate consumption

**Figure 1:**
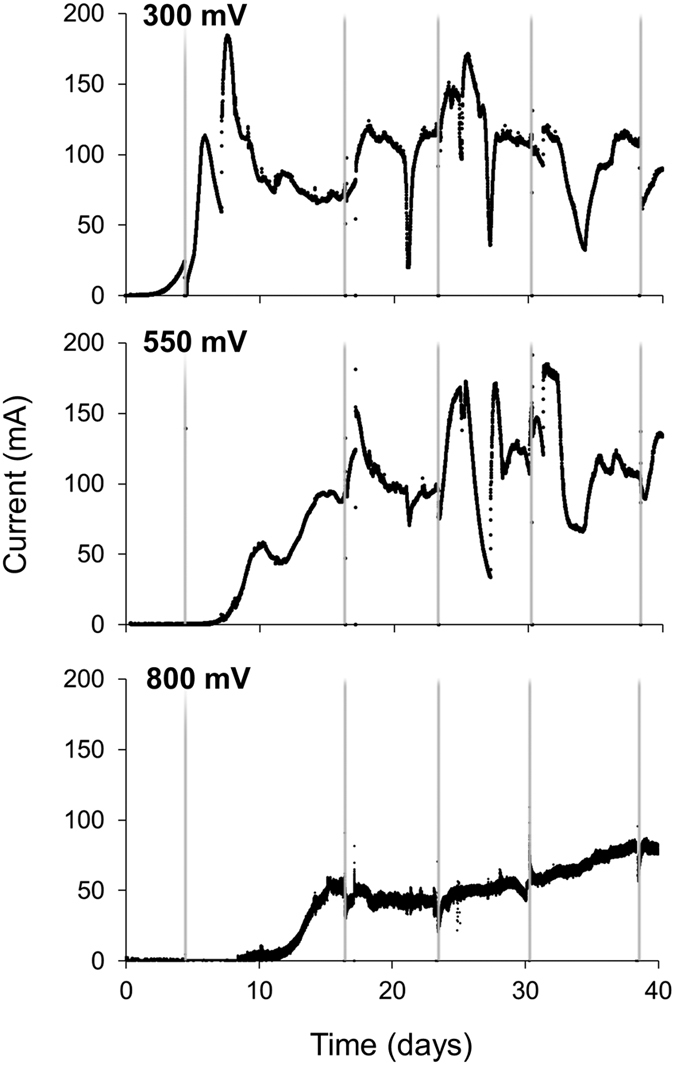
Chronoamperometric profiles at different anode potentials. The chronoamperometric output was from all 15 anodes within each reactor. The grey vertical lines indicate the times that the reactors were paused for biofilm sampling.

Chronoamperometry revealed that all reactors developed catalytic activity within the first 15 days after inoculation (Fig. 1). The time needed for current onset was positively associated with anode potential (*P* < 0.001; Fig. 1). Throughout the experiment, current generation was more stable but significantly lower (*P* < 0.001) at 800 mV than at 550 and 300 mV (Fig. 1). This is also reflected by the cumulative charge, which was significantly less for the 800 mV reactor than for those held at 550 and 300 mV (Table S1, Fig. 4B). Over the duration of the experiment, under potentiostatic conditions, more current was generated at 300 mV than at 550 mV (*P* < 0.001), although there were instances when the 550 mV reactor outperformed the 300 mV reactor (Fig. 1). This is confirmed by the larger cumulative charge of the 300 mV reactor compared to that at 550 mV (Table S1).

**Figure 2:**
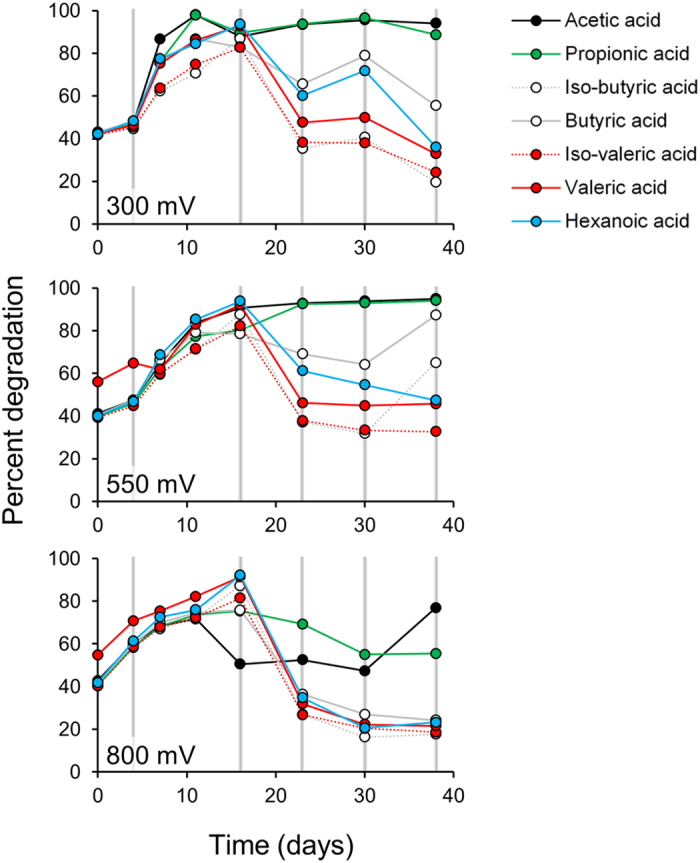
Degradation of Volatile Fatty Acids at different anode potentials over time. The grey vertical lines indicate the times that the reactors were paused for biofilm sampling.

Volatile fatty acid (VFA) degradation profiles differed significantly over time (*P* < 0.001). Initially, the consumption of VFAs occurred evenly and increased until day 16 after which, degradation rates of all VFAs other than acetate and propionate declined (Fig. 2). Anode potential did not significantly influence the types of VFAs utilized, but a lesser quantity of VFAs were degraded at 800 mV than at 300 mV and 550 mV (*P* < 0.05).

### Cyclic voltammetry and biofilm characterisation

**Figure 3:**
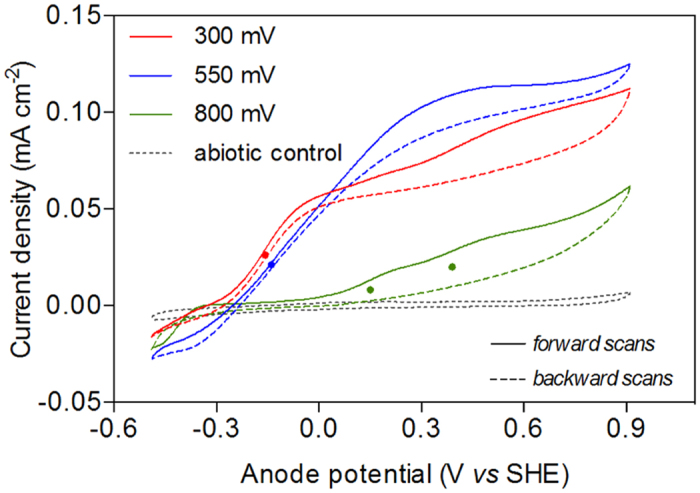
Representative turnover cyclic voltammograms at a scan rate of 1 mV s^−1^ of biofilms metabolizing volatile fatty acids. Red, blue, and green lines indicate CVs performed on the BES operated at 300 mV, 550 mV, and 800 mV, respectively. Solid lines indicate forward scans (from negative to positive potentials) and dashed lines indicate backward scans (from positive to negative potentials). Dotted line indicates a CV measured on a sterile electrode immersed in the same electrolyte as the other BESs. Putative electron transfer sites (E_f_) were determined by first derivative analysis (FDA) and are indicated with dots in the CVs. E_f_ were centered at −158 mV and at −140 mV for the BES operated at 300 mV and 550 mV, respectively. The BES operated at 800 mV displayed a less clear current vs potential response with two putative electron transfer sites centered at +150 mV and +390 mV.

The response of current to anode potential for electrode-associated biofilms was assessed by cyclic voltammetry in the presence of metabolic electron donors (Fig. 3). Measurements performed on abiotic control electrodes (Fig. 3) show that no appreciable current was detected, indicating that biofilms in all systems were responsible for promoting electron transfer to the anodes. Cyclic voltammetry was performed on a single electrode from each reactor after disconnecting each from the parallel circuits. Biofilms associated with the 300 mV and 550 mV produced appreciable current at potentials above approximately −0.3 mV. Current then started to increase rapidly around the midpoint potentials of c. −158 mV and −140 mV (*i.e.* the formal potential *E*_f_ of the dominant electron-transfer site as identified by analysis of the first derivative traces, not shown) for reactors at 300 mV and 550 mV, respectively. Beyond the *E*_f_, the sharp current response to applied potential ceased. However, while the current profile for the 550 mV reactor reached a plateau at higher potentials, indicating that additional increases of the driving force does not result in faster reaction rates, the current profile of the 300 mV reactor showed a residual slope at potentials between 0 mV and the upper limit of 910 mV, indicating that additional putative redox-active species were activated in the high-potential range. A similar trend at high potentials is also observed for the BES operated at 800 mV. However, this reactor lacked the initial steep current response that was observed in the other reactors. The 800 mV reactor started producing appreciable current only at potentials above 0 mV, which is higher than the onset observed in the other reactors. Nonetheless, rather than increasing sharply around a midpoint potential as observed in the reactors, the current increased more gradually with applied potential. This gradual rise in current may be indicative of an electron transfer process characterized by a slow interfacial electron transfer rate constant. Analysis of the first derivative traces highlights the presence of two less distinctive putative redox-active centers at the potentials of approximately +150 mV and +390 mV.

**Figure 4:**
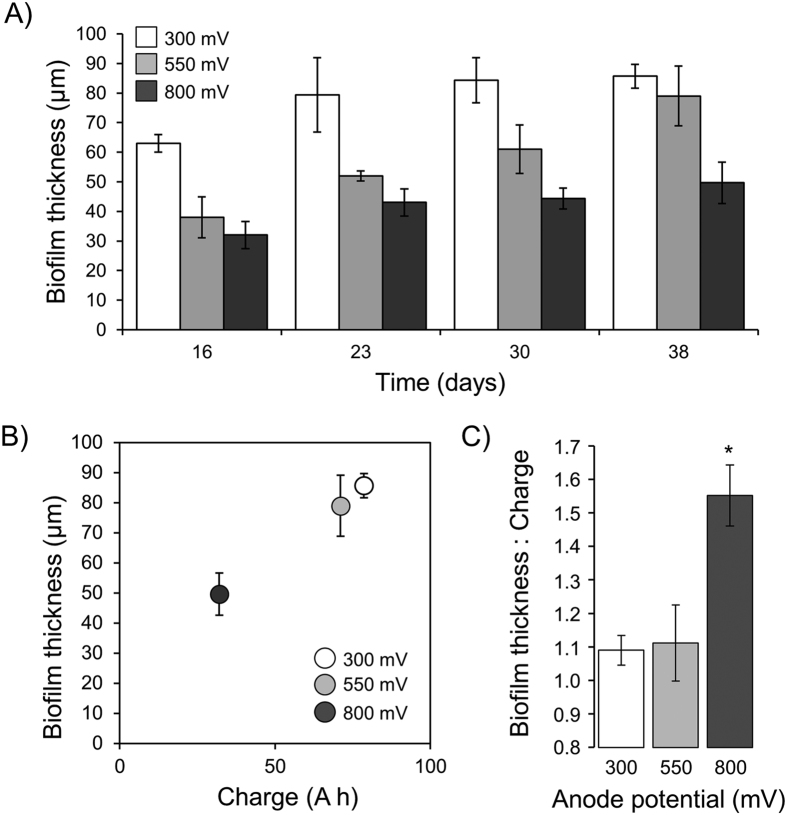
(**A**) Anodic biofilm thickness at different potentials (SHE) over time. (**B**) Anodic biofilm thickness as a function of charge after 38 days at different potentials. (**C**) Biofilm thickness per unit charge after 38 days at different potentials. Error bars represent standard deviations and the asterisk in panel C signifies that significantly (P = 0.03) more biofilm thickness was produced per unit charge at 800 mV than at 300 mV or 550 mV.

Biofilm thickness increased significantly over time (*P* = 0.002; Fig. 4A), and was negatively associated with anode potential (*P* < 0.001; Fig. 4B) with the 800 mV reactor showing significantly lower overall thickness (Fig. 4). Despite this, when expressed as a ratio per unit charge, biofilms at low potentials were of similar thickness while those at 800 mV were significantly thicker (Fig. 4C; *P* < 0.03).

### Microbial diversity

**Figure 5:**
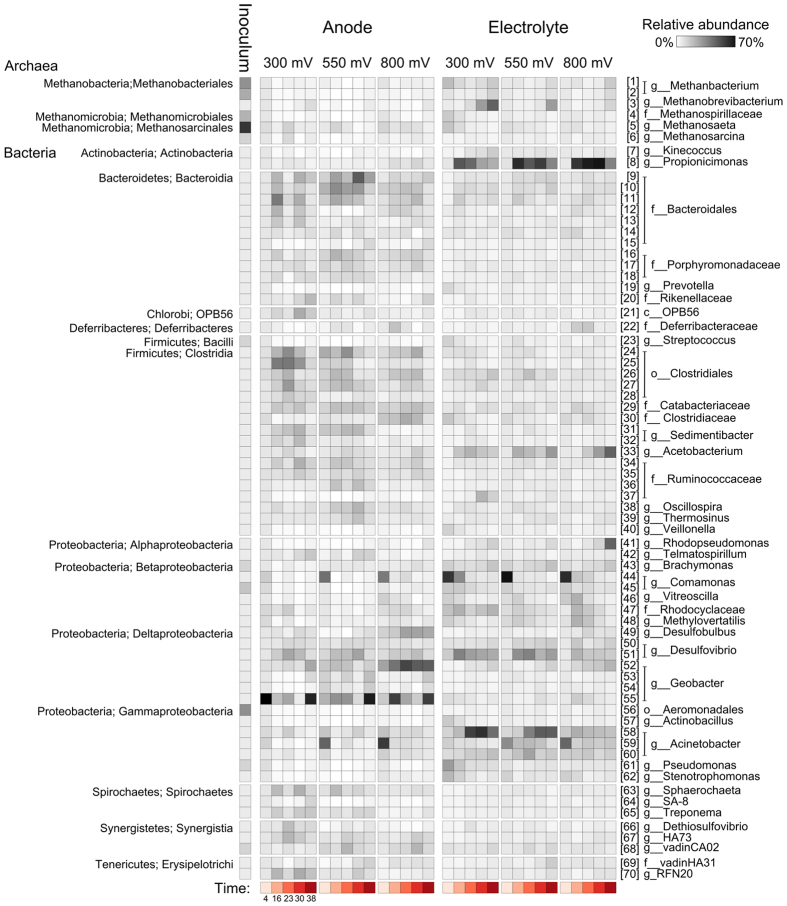
Heatmap summarizing the abundances of OTUs that were present at more than 2% in at least one sample. All abundances are Hellinger transformed and represent the mean of three replicates. The numbers in brackets are OTU ids and are consistent with other figures.

The composition of inoculum, anode and electrolyte communities differed significantly (Fig. 5) (P < 0.001 for Hellinger transformed OTU abundances and weighted unifrac distances), with larger abundances of several archaeal populations in the inoculum, an enrichment of *Geobacter* populations on the anodes and more abundant *Propionicomonas, Acinetobacter* and *Acetobacterium* populations in the electrolyte (Figs 5, S3). Relative to the inoculum, microbial communities in the reactors had fewer OTUs and lower Faith’s PD (*P* < 0.03), but similar Simpson’s Diversity Index values (Fig. S4).

**Figure 6:**
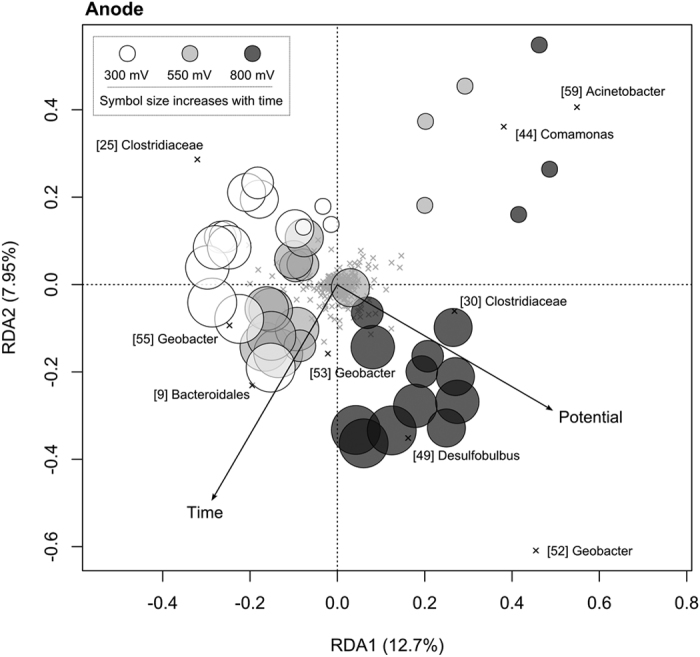
Redundancy analysis ordination summarizing variation in the composition of microbial communities over time (circle size increases with time) between anodes set at different potentials. OTUs are shown as crosses and those that discriminate between treatments are labelled consistently with the heatmap and other figures.

**Figure 7:**
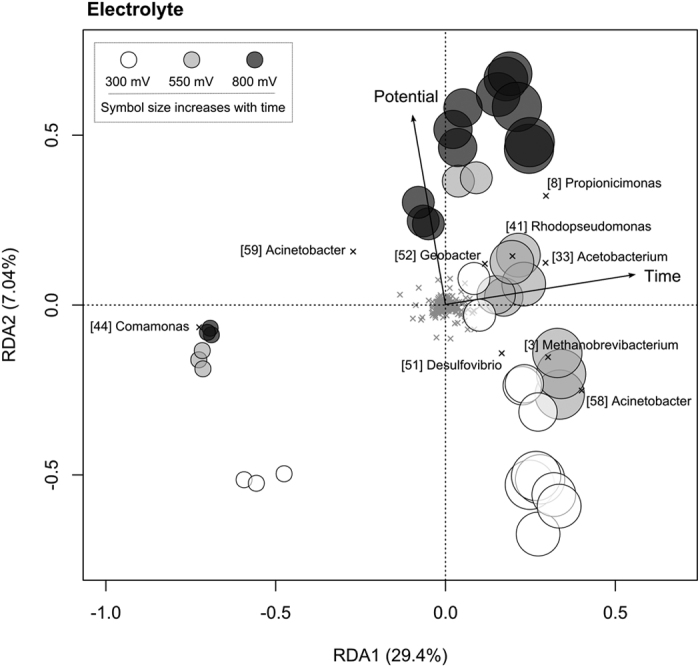
Redundancy analysis ordination summarizing variation in the composition of microbial communities associated with the electrolyte of anodes set at different anode potentials over time. OTUs are shown as crosses and those that discriminate between treatments are labelled consistently with the heatmap and other figures.

**Figure 8:**
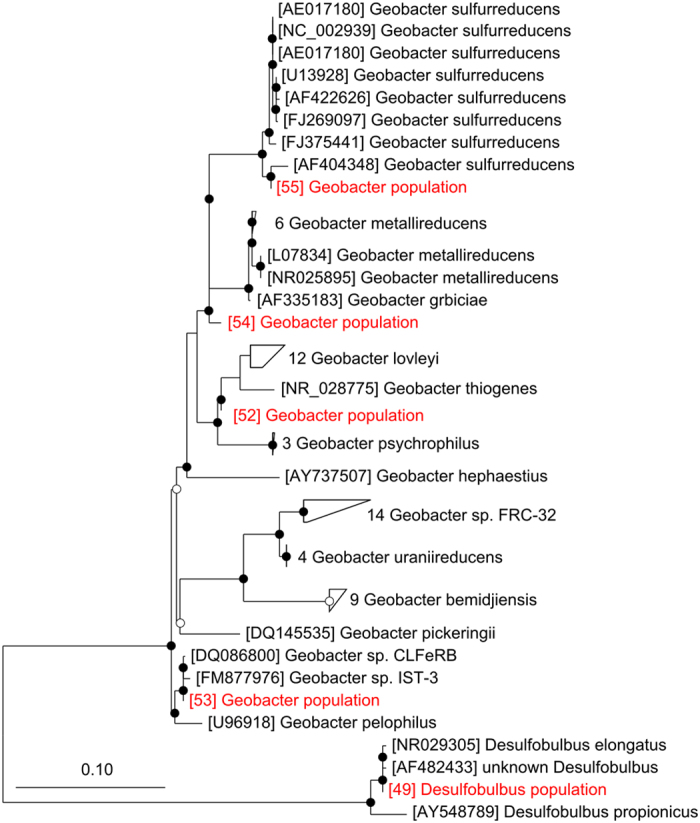
Phylogenetic neighborhood of selected OTUs.

Within the reactors, the composition of both anodic electrode (Fig. 6) and electrolyte (Fig. 7) associated microbial communities was influenced by anode potential and time (*P* < 0.001). For electrode-associated communities, anode potential and time explained 9.9% and 9.5% of compositional variation, respectively (*P* < 0.001). Most of the variance attributed to time, however, was associated with the onset of current generation and the enrichment of *Geobacter* populations, such as OTU 55, which is a close relative of *Geobacter sulfurreducens* (Figs 5, 6 and 8). Electrode communities held at 300 mV, for example, were dominated by OTU 55 in all time points and current was generated prior to the first sample collection (Fig. 1). In contrast, electrodes held at 550 and 800 mV took significantly longer to develop current and the enrichment of OTU 55 was not detected until the second sampling (16 days; Fig. 5). At four days, the electrocatalytically inactive electrodes held at 550 and 800 mV were dominated by *Acinetobacter* (OTU 59) and *Comamonas* (OTU 44) populations, which were also common in the electrolyte (Fig. 5). From days 16 until 38, a period in which all reactors were generating current, anode potential and time explained 14.7% and 9.0% of composition variation in electrode-associated microbial communities, respectively (*P* < 0.001).

The most pronounced effect of anode potential on the composition of electrode-associated communities was the enrichment of a distinct *Geobacter* representative (OTU 52) and a *Desulfobulbus* population (OTU 49) at 800 mV, relative to lower reduction potentials (Figs 5 and 6; *P* < 0.001). In all reactors, several *Bacteroidetes* and *Clostridiales* populations initially increased in abundance, but then declined from 23–30 days (Fig. S5).

For electrolyte-associated communities, anode potential and time explained 8.52% and 23.2% of compositional variation, respectively (*P* < 0.001). Electrolyte-associated communities were initially dominated by a *Comamonas* population (OTU 44) that rapidly declined in all reactors (Figs 5 and 7). In reactors held at 550 and 800 mV, an *Acinetobacter* population (OTU 59) also represented a dominant component of the electrolyte communities (Fig. 5). By Day 16, the electrolyte of all reactors was dominated by populations belonging to the genera *Propionicimonas* (OTU 8), *Acetobacterium* (OTU 33) and *Desulfovibrio* (OTU 51) (Figs 5, S5). Over time differences were also apparent between reactors held at different anode potentials. At 300 and 550 mV, the anolyte became enriched with an *Acinetobacter* population (OTU 58) and a representative of the archaeal genus *Methanobrevibacterium* (OTU 3) (*P* < 0.01). At 800 mV, the anolyte became enriched with a *Geobacter* population (OTU 52) and by Day 38 a member of the genus *Rhodopseudomonas* (OTU 41) was also abundant (*P* < 0.01) (Fig. S5). Despite being one of the dominant populations in all reactors the *Acetobacterium* (OTU 33) was also more abundant at 800 mV than at lower potentials (*P* < 0.001).

At Day 7, anodic electrode associated microbial communities at 300 mV were consistently more diverse that those at 550 mV and 800 mV, while in the electrolyte, microbial diversity at 300 mV was lower than at 550 mV and 800 mV (*P* < 0.05; Fig. S4). After Day 7, however, the alpha diversity of anodic electrode and electrolyte associated microbial communities, as indicated by the number of observed OTUs, Simpson’s Diversity Index and Faith’s PD, did not exhibit any association with time or potential (Fig. S4).

## Discussion

According to thermodynamics, microorganisms should obtain more energy when coupling respiration to more oxidising anodes as long as they can harvest the energy via generation of ATP by producing additional proton motive force^3^. Based on this rationale, we tested the hypotheses that anode potential is positively associated with microbial biomass and activity, and influences microbial diversity.

Our results indicated significant effects of anode potential on microbial biomass, activity and diversity. Contrary to our hypotheses, however, microbial biomass (*i.e.* biofilm thickness) and activity (*i.e.* VFA degradation and average current) were significantly lower at the highest anode potential. On face value these results suggest that anode-respiring microorganisms obtain less energy at high potentials. However, relative to the total charge generated, more biomass was produced at high potential than at lower potentials (Fig. 4B,C). This indicates that despite less charge passing through biofilms at high potential, microorganisms were able to obtain a larger proportion of this energy for growth.

Using turnover CV and first derivative analysis, we detected the presence of two putative electron-transfer sites in biofilms maintained at 800 mV that had higher mid-point potentials than that of the putative redox-active species detected in the 300 and 550 mV reactors. This may indicate that the biofilms at 800 mV harbored microorganisms with terminal reductases that facilitated greater energy gain per unit charge than those at lower potential.

A question remains, though, as to why the biomass and activity of microbial communities at high potential was less than that at lower potential. One possibility is that organisms capable of performing extracellular electron transfer are better adapted to the reduction of low potential electron acceptors, in which case our +800 mV anodes would have been an anomaly. Another explanation is that biofilm growth and activity were limited at high potential by oxidative stress at the electrode surface. Electrochemical oxygen evolution at the high potential of 800 mV can most likely be excluded in our system, as also shown by the negligible catalytic wave produced by the abiotic control electrode in the range of potentials used during the CV tests (Fig. 3). High electrode potentials (+597 to +797 mV *vs* SHE) have previously been shown to reduce current production and degrade proteins in *Shewanella oneidensis* MR-1^14^ and *Geobacter sulfurreducens*^15^.

Current evidence indicates that *G. sulfurreducens* biofilms go through an initial phase where cells need to optimize attachment or electron transfer to the electrode. After this phase, the rate that cells can deliver electrons to their external surfaces limits current generation relative to the transfer of electrons between cells and to electrodes^16^. It is possible, therefore, that the relatively stressful conditions at high potential would have constrained this initial attachment phase due to denaturation of outer membrane cytochromes as suggested by TerAvest and Angenent^14^. An increase in the detachment rate of cells from the anode surface has been identified as a key factor contributing to reduced biofilm thickness in model simulations^17^. This could plausibly explain why microorganisms that are apparently more energy efficient were unable to generate more charge and attain greater biofilm thickness.

The results of our study indicate that anode potential significantly influences the composition of anodic electrode and electrolyte associated microbial communities. This highlights that measured differences in reactor performance between anode potentials may be related to both structural and/or physiological changes in the community. Our findings contradict those of Zhu *et al*.^4^, but support other studies concerning the relationship between anode potential and microbial community composition^5^,^8^,^12^. Furthermore, as our reactors facilitated triplicate sampling of anodes over time, our results reveal that the influence of anode potential on microbial community composition was apparent throughout the course of the experiment. It is important to consider, however, that as with previous studies^4^,^5^,^8^,^12^, our observations may be specific to the inoculum that we used.

One aspect of community ecology that has been largely overlooked in MFCs is the relationship between anode potential and alpha diversity (i.e. the number of taxa (richness) and the similarity of their abundances (evenness)). To our knowledge the only study to report such data is that of Torres *et al*.^8^ in which a strong positive association was observed between anode potential and the number of microbial taxa. Our results indicate that anode potential exerted negligible effects on alpha diversity in all but the first sampling date (Fig. S4). Critically, we did not detect a positive correlation between alpha diversity and anode potential as observed by Torres *et al*.^8^.

Another aspect of community ecology that has been largely overlooked in MFCs is the response of anodic electrolyte associated microbial communities to different anode potentials. As previously observed, the diversity of the inoculum microbial community changed rapidly upon introduction to the reactors, reflecting the highly selective nature of MFC environments^5^,^13^,^18^. With the onset of current generation, the composition of anodic electrode and electrolyte associated communities significantly diverged (Fig. S3). Anodes became dominated by OTUs closely related to bacteria that are able to perform extracellular electron transfer (e.g. *Geobacter* populations) while electrolyte-associated communities were dominated by fermentative organisms (e.g. members of the *Propionicimonas, Acetobacterium* and *Acinetobacter*^19^).

Anode potential influenced the diversity of both anodic electrode and electrolyte associated communities. In the absence of current generation, reactors were dominated by an *Acinetobacter* and a *Comamonas* population. These OTUs rapidly declined with increasing current, even in the electrolyte where they were replaced by a *Propionicimonas* population, a member of the *Acetobacterium* and a representative of the *Desulfovibrio*. These populations may have simply been more tolerant to oxidative stress; however, their positive association with current may indicate a more direct role in electron transfer. For example, *Acetobacterium* spp. often dominate mixed acetogenic communities on biocathodes^20^,^21^ and may, therefore, engage in extracellular electron transfer with anodic electrode-associated cells. In addition, members of the *Desulfovibrio* have been shown to generate current in mediator-less MFCs^22^.

While anodes at all potentials were dominated by an OTU closely related to *Geobacter sulfurreducens* (OTU 55), at 800 mV there was an enrichment of a different *Geobacter* population (OTU 52) and a member of the *Desulfobulbus* (OTU 49). The *Desulfobulbus* population was closely related to *D. elongatus* and *D. propionicus*, the latter of which is known to be capable of reducing insoluble electron acceptors such as electrodes^23^ (Fig. 8). The distinct *Geobacter* population (OTU 52) was closely related to *G. thiogenes* and *G. lovleyi* (Fig. 8). It is possible that the *Geobacter* and *Desulfobulbus* spp. enriched at 800 mV harboured the putative redox-active species with higher mid-point potentials as detected by turnover CV. This may have given them access to greater potential energy gain and therefore a competitive advantage at high potential. Interestingly, in a comparative genomics study of six *Geobacter* genomes including *G. sulfurreducens* and *G. lovleyi*, Butler *et al*.^24^ found that all genomes contained a large number of cytochrome-encoding genes but that these were poorly conserved between species. *G. lovleyi* had the least cytochrome-encoding genes and a genome-based phylogeny revealed that it is distinct from other geobacter genomes^24^. An independent 16 S rRNA gene-based phylogeny of *Geobacter* spp. also highlights that *G. lovleyi* is phylogenetically distinct from most representatives of the genus^25^. As suggested by Commault *et al*.^12^, differences in reactor performance between anode potentials may reflect the selection of *Geobacter* populations (and close relatives) with differing traits, rather than solely changes in the physiology of dominant populations as indicated by Zhu *et al*.^4^.

Our results show clear effects on microbial diversity and indicate that biofilms at +800 mV generated more biomass per unit charge than those at lower potential. This suggests that the organisms may be capable of obtaining more energy when coupling respiration to high potential electron acceptors. However, throughout the experiment, biofilms at +800 mV were thinner, transferred less charge and oxidized less substrate than those at lower potentials. This suggests that, in our systems, organisms capable of extracellular electron transfer were not able to capitalize on the potential for additional energy gain due to a lack of adaptive traits to high potential electron acceptors and/or were sensitive to oxidative stress.

## Materials and Methods

### Reactor setup

Three lamellar type reactors, each consisting of two end plates and five anode-cathode compartments were constructed from acrylic sheeting as described in Dennis *et al*.^13^ (Fig. S1). Each anode frame housed three 4 × 30 × 150 mm IGS-743 graphite blocks serving as working electrodes (Morgan Industrial Carbon, Australia), which were mounted in acrylic blocks with rubber o-rings leaving 120 mm electrode length exposed to the medium. Each reactor contained 15 electrodes so the total electrode surface area was 1242 cm^2^ (540 cm^2^ projected relative to membrane).

Anode and cathode compartments were separated by a cation exchange membrane (Ultrex CMI7000, Membranes International Inc., USA) sandwiched between two 2 mm thick rubber frames with the same dimensions as the electrode frames. Each cathodic electrode consisted of a stainless steel wire mesh (316 SS, size 300, Locker, Australia) in a stainless steel frame. Anode-cathode pairs were separated by a sheet of impermeable rubber. When all of the layers were correctly positioned, the end plates were fastened in place and each reactor was connected to its own set of recirculation and buffer vessels. To maximize consistency between reactors the feed came from one vessel. The total liquid volume of the anode and cathode circuits was 2.8 and 2.9 L, respectively.

All 15 anodic electrodes per reactor were connected in parallel to a potentiostat (Potentiostat/Galvanostat VMP3, BioLogic Science Instruments, France). A Ag/AgCl electrode filled with 3 M KCl (+210 mV *vs* SHE) was fitted to the middle anode compartment of each reactor via a glass capillary ending in a glass frit and served as reference electrode. All potentials hereafter are reported with respect to the SHE. Anodic current and cumulative charge were monitored using the potentiostat and were processed using the EC-Lab^®^ V9.2 software package (Bio-Logic-SAS, France). A pH electrode was fitted to the anodic recirculation circuit of each reactor and attached to a control module that activated a 1 M NaOH dosing pump. These systems were set to maintain anodes at pH 6.5.

### Reactor operation

Each reactor was seeded with a diverse microbial community originating from a mixture of eight anaerobic environments: three mesophilic anaerobic digesters, a thermophilic anaerobic digester, an upflow anaerobic sludge bioreactor, an anaerobic lagoon, rumen contents and lake sediment. The inoculum contained an equal quantity of volatile suspended solids from each source and has been characterized previously^26^. The reactors were operated in potentiostatic mode at a potential of 300 mV, 550 mV and 800 mV, respectively. The anode compartments were fed a mixture of volatile fatty acids in modified M9 medium and 1 ml l^−1^ of a mixed trace element solution (Supplementary information). The cathode compartment was fed 1 g l^−1^ NaCl. The feed rate for both electrolytes was 1.42 ± 0.04 l d^−1^. This led to an organic loading rate of 1.57 g fatty acids l^−1^_anode_ d^−1^ or 1.7 g COD l^−1^_anode_ d^−1^.

### Electro-catalytic activity

Cyclic voltammetry (CV) was performed under turnover conditions (i.e. in the presence of metabolic substrates) on anodic electrodes after 50 days of operation. During the measurement, the potential of the working electrodes (the anodes) was swept between −490 mV and +910 mV *vs* SHE at a scan rate of 1 mV s^−1^. Control measurements were obtained from sterile anodes while soaked in the same electrolyte as the reactors, in order to mimic the chemical environment within the anode compartment. The inflection points of catalytic waves were identified by plotting the derivative of each sweep as a function of the potential (*i.e.*, first derivative analysis). This analysis facilitated identification of the formal potential (*E*_*f*_) of putative catalytic moieties.

### VFA utilisation profiles

Liquid samples obtained from the anode and cathode compartments were filtered through 0.22 μm sterile filters and then volatile fatty acid (VFA) content and chemical oxygen demand (COD) were determined as described in the Supplementary Information.

### Microbial diversity: Sample collection and DNA extraction

At 4, 16, 23, 30 and 38 days operation anodic biofilms and electrolyte samples were taken in triplicates to characterize the diversity of microbial communities. On each sampling occasion the recirculation streams were shut-down and the potentiostat was paused. All three electrodes associated with a reactor compartment were then removed and replaced with sterile electrodes. A 1 ml sample of the anolyte was centrifuged at 4,000 g for 5 min and the pellet was stored at −80 °C until further analysis. Biofilms were collected from the surface of the anodic electrodes using sterile glass microscope slides, snap frozen in liquid nitrogen and transferred to −80 °C stored until further processing. A section of each electrode was also snapped-off for measurement of biofilm thickness. DNA was extracted from the anolyte pellet and electrode biofilms using a MO BIO PowerBiofilm™ DNA Isolation Kit according to the manufacturer’s instructions.

### PCR amplification and pyrosequencing

Universal 16 S rRNA genes were amplified by PCR, purified and then pooled for 454 pyrosequencing as described in the Supplementary Information. Sequencing was performed by Macrogen Inc. (Seoul, Korea).

### Analysis of sequence data

Sequences were quality filtered and dereplicated using the QIIME script split_libraries.py with the homopolymer filter deactivated^27^ and then checked for chimeras against October 2013 release of the GreenGenes database^28^ using UCHIME^29^ ver. 3.0.617. Homopolymer errors were corrected using Acacia^30^. Sequences were then subjected to the following procedures using QIIME: 1) sequences were clustered at 97% similarity using UCLUST^31^, 2) GreenGenes taxonomy (October 2013 release) was assigned to the cluster representatives using BLAST, and 3) tables with the abundance of different OTUs and their taxonomic assignments in each sample were generated. In addition, full length sequences that were identified as the nearest BLAST matches for each OTU were aligned and a midpoint rooted phylogenetic tree was generated. The number of reads was then rarefied to 500 sequences per sample. The mean number of OTUs, Simpson’s Diversity Index values and Faith’s Phylogenetic Diversity Index values were calculated using QIIME. Variation in the composition of microbial communities between normalized samples was investigated from a taxonomic (relative OTU abundances) and phylogenetic (weighted unifrac distances) perspective^32^. The phylogenetic neighborhood of selected OTUs was determined by aligning their sequences with the October 2013 release of the GreenGenes database using PyNAST^33^. All sequences were then inserted into the full GreenGenes phylogeny by maximum parsimony using Arb^34^.

### Biofilm thickness

Fluorescent *in-situ* hybridisation (FISH) was performed as described by Amann *et al*.^35^. Anodes were fixed in 4% paraformaldehyde (PFA) overnight at 4 °C, washed twice with 1X PBS and then stored in 1:1 PBS and ethanol mixture at −20 °C. Fixed samples were dehydrated for three minutes in 50%, 80% and 98% ethanol, respectively, and then hybridized with a Fluorescein isothiocyanate (FITC)-labelled bacterial probe EUB338^36^ (5′- GCTGCCTCCCGTAGGAGT -3′) for two hours at 46 °C. Post hybridization, samples were washed with standard FISH wash-buffer at 48 °C for 15 min, desalted by immersion in 4 °C water for 2–3 secs and then dried. Samples were mounted with an anti-bleaching agent 1,4-diazabicyclo[2.2.2]octane (DABCO, Sigma, Australia) before being viewed with a LSM 510 confocal laser scanning microscope (Zeiss, Germany) equipped with an argon (488 nm) laser. Images were captured and visualized by LSM AIM 4.2 (Zeiss, Germany) at 630X magnification. An average of three scans from each biofilm was taken to measure thickness. Biofilm thickness was measured via 3D reconstruction of z-stack images scanned at 3 μm intervals from the top of the biofilms to the anode surface using LSM AIM 4.2 (Zeiss, Germany). Biofilm structure and mean thickness was quantified using COMSTAT image analysis software, using connected volume filtration to reduce background noise^37^.

### Statistical analyses

The influence of time and potential on univariate response variables, such as current, start-up time, total VFAs degraded, alpha diversity metrics and biofilm thickness, were analysed using linear regression and/or analysis of variance. The influence of these parameters on multivariate responses, such as substrate (i.e. VFA) utilisation patterns, OTU abundances and unifrac distances were assessed using permutational multivariate analysis of variance (PERMANOVA). Redundancy Analysis (RDA) was used to highlight populations that discriminated between communities separated by potential and time. All analyses were performed using R.

## Additional Information

**How to cite this article**: Dennis, P. G. *et al*. Anode potential influences the structure and function of anodic electrode and electrolyte-associated microbiomes. *Sci. Rep.* **6**, 39114; doi: 10.1038/srep39114 (2016).

**Publisher's note:** Springer Nature remains neutral with regard to jurisdictional claims in published maps and institutional affiliations.

## Supplementary Material

Supplementary Information
