# Supplementary material for: Anode potential influences the structure and function of anodic electrode and electrolyte-associated microbiomes
Source: Sci Rep. 2016 Dec 19;6:39114. doi: 10.1038/srep39114 (PMC5171916; doi:10.1038/srep39114)
Supplement: Supplementary Information [file srep39114-s1.pdf]

Supplementary Information:

Anode potential influences the structure and function of anodic electrode and electrolyte-associated microbiomes

Paul G. Dennis[1,2,3], Bernardino Virdis[2,4], Inka Vanwonterghem[2,3], Alif Hassan[2,3], Phil Hugenholtz[3], Gene W. Tyson[2,3*], Korneel Rabaey[2,5*]

*[1]School of Agriculture and Food Sciences, The University of Queensland, Brisbane, Queensland 4072, Australia; [2]Advanced Water Management Centre, The University of Queensland, Brisbane, Queensland 4072, Australia; [3]Australian Centre for Ecogenomics, The University of Queensland, Brisbane, Queensland 4072, Australia; [4]Centre for Microbial Electrochemical Systems, The University of Queensland, Brisbane, Queensland 4072, Australia; [5]Laboratory of Microbial Ecology and Technology, Ghent University, Coupure Links 653 9000 Ghent, Belgium; *email:* Korneel.Rabaey@UGent.be, g.tyson@uq.edu.au

Contents:

Fig. S1……………………………………………………………………………………2

Fig. S2……………………………………………………………………………………3

Fig. S3……………………………………………………………………………………4

Fig. S4……………………………………………………………………………………5

Fig. S5……………………………………………………………………………………6

Table S1…..…………………………………………………………………………...7

Supplementary methods………………………………………………………………….8-9

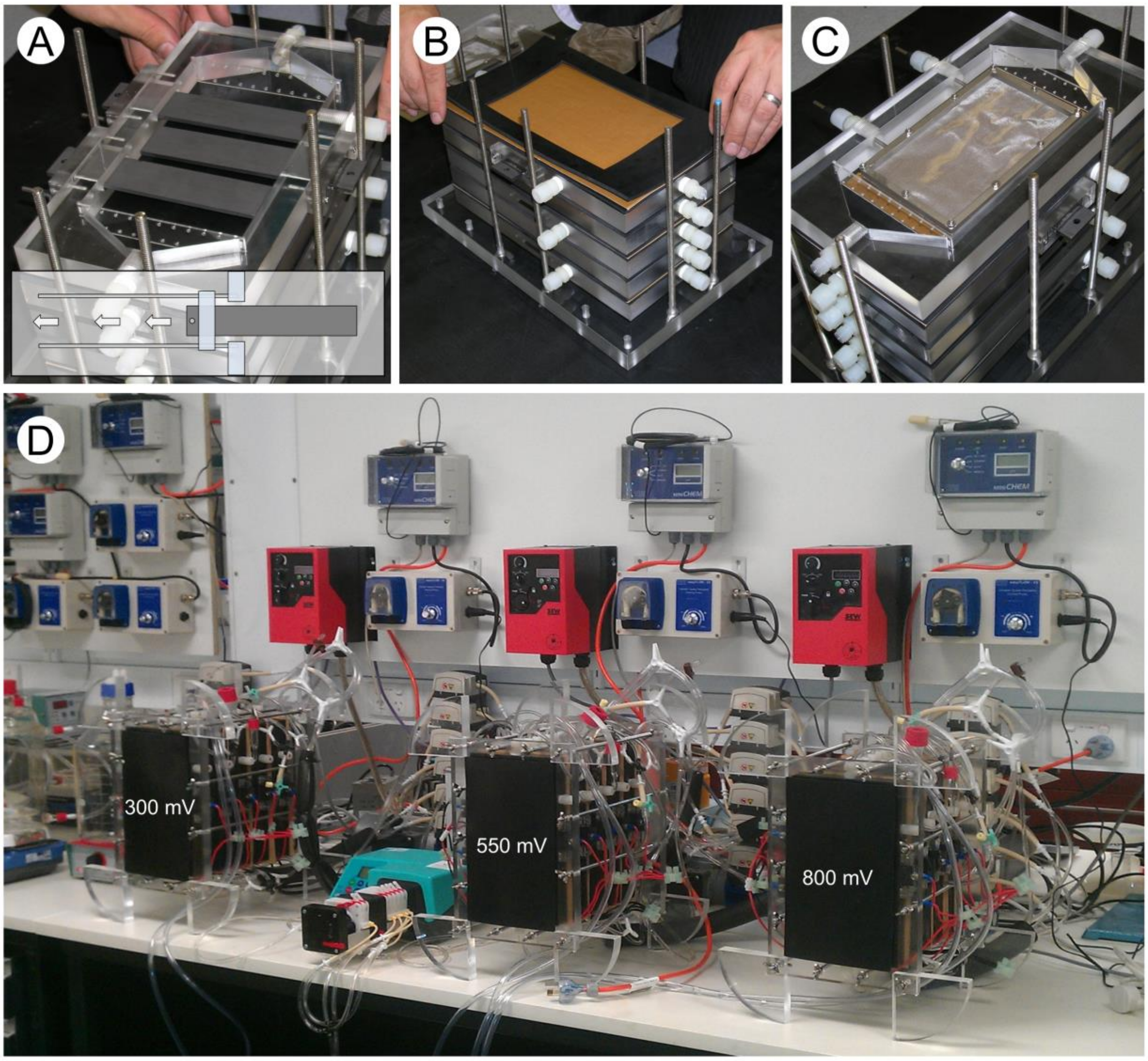

Fig. S1 Photographs of the reactor setup: (A) an anode frame fitted with three exchangeable anodic electrodes; (B) anode and cathode compartments were separated by a cation exchange membrane sandwiched between two rubber frames; (C) a cathode frame fitted with an assembled cathodic electrode; (D) an overview of the assembled reactors showing pH control and recirculation pumps.

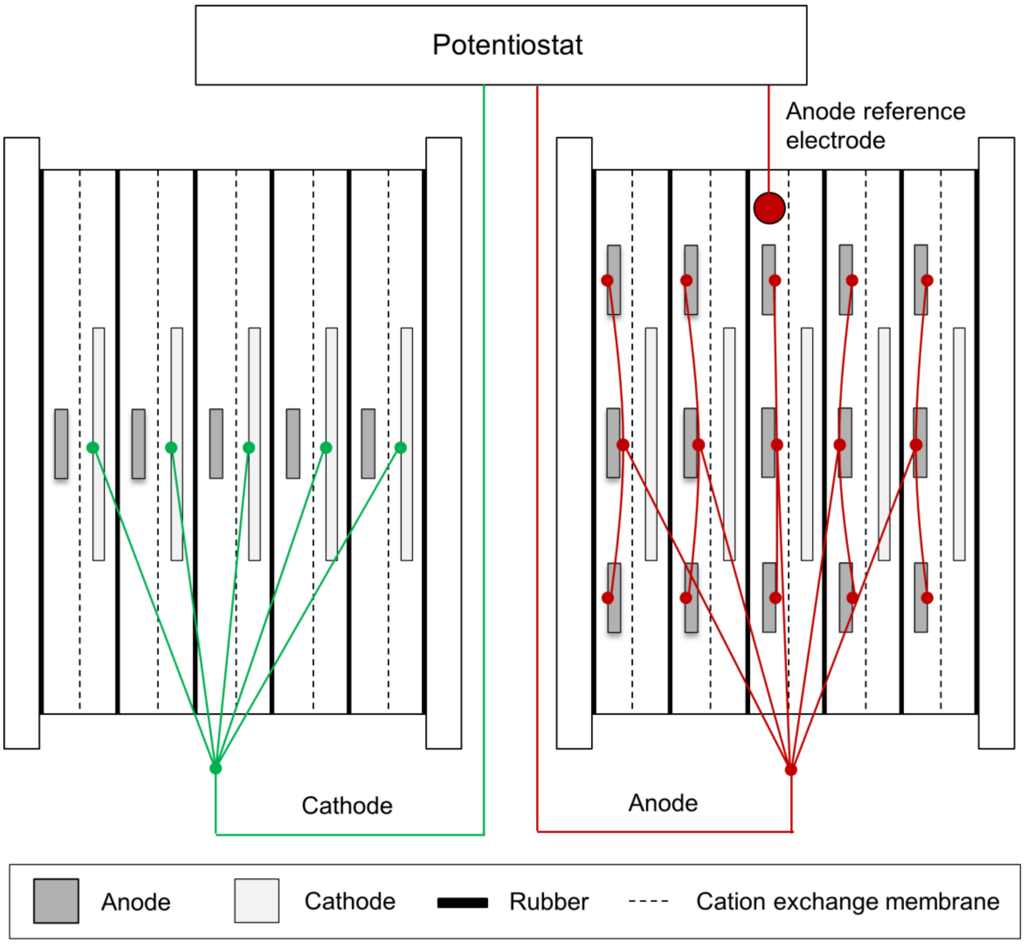

Fig. S2 Schematic diagram of the electrical connections highlighting that all 15 anodic electrodes and all five cathodic electrodes per reactor were connected.

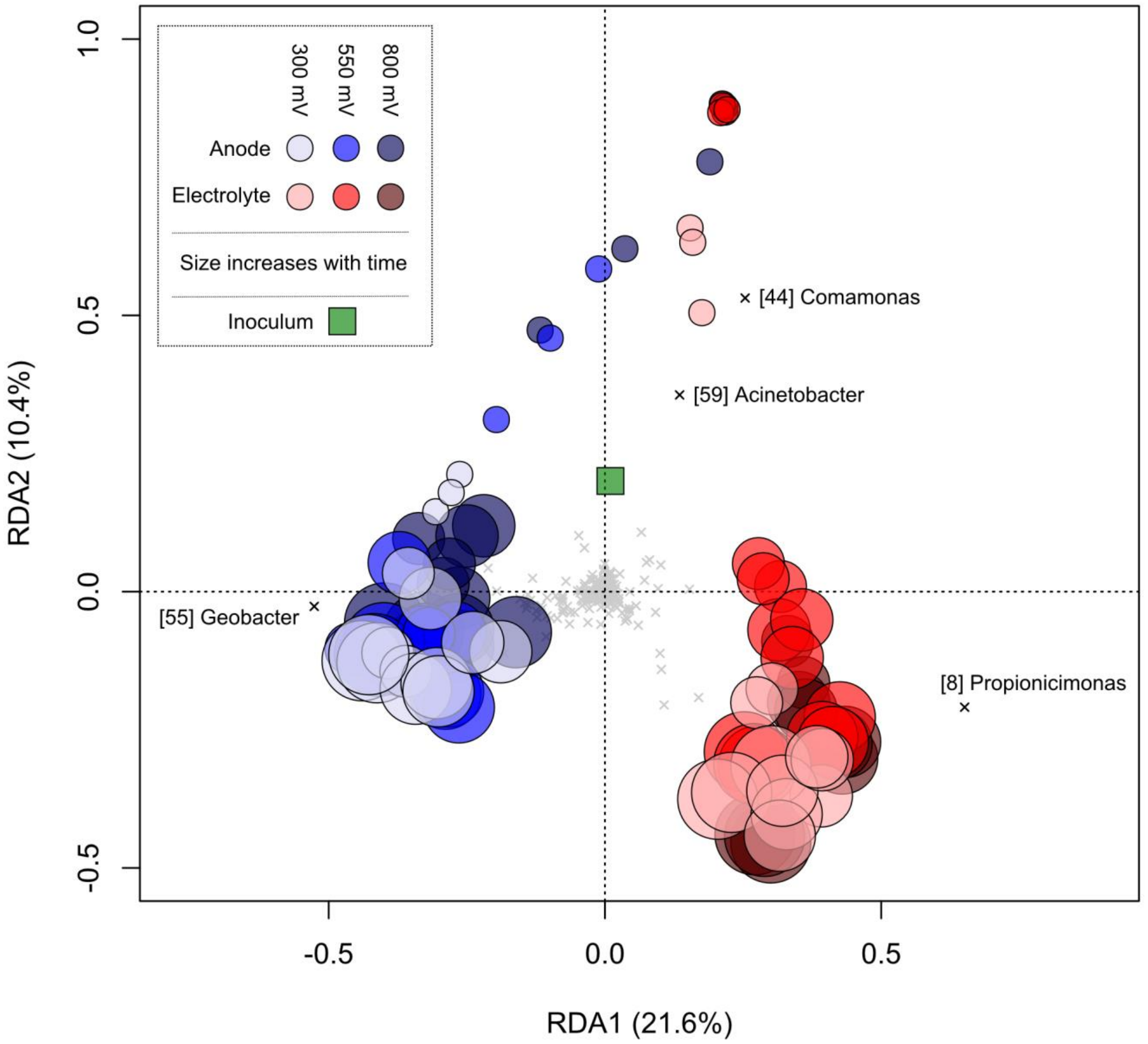

Fig. S3 Redundancy analysis ordination summarising differences in the composition of microbial communities between the inoculum and the reactor-associated samples over time. OTUs are shown as crosses and those that discriminate between treatments are labelled consistently with the heatmap and other figures.

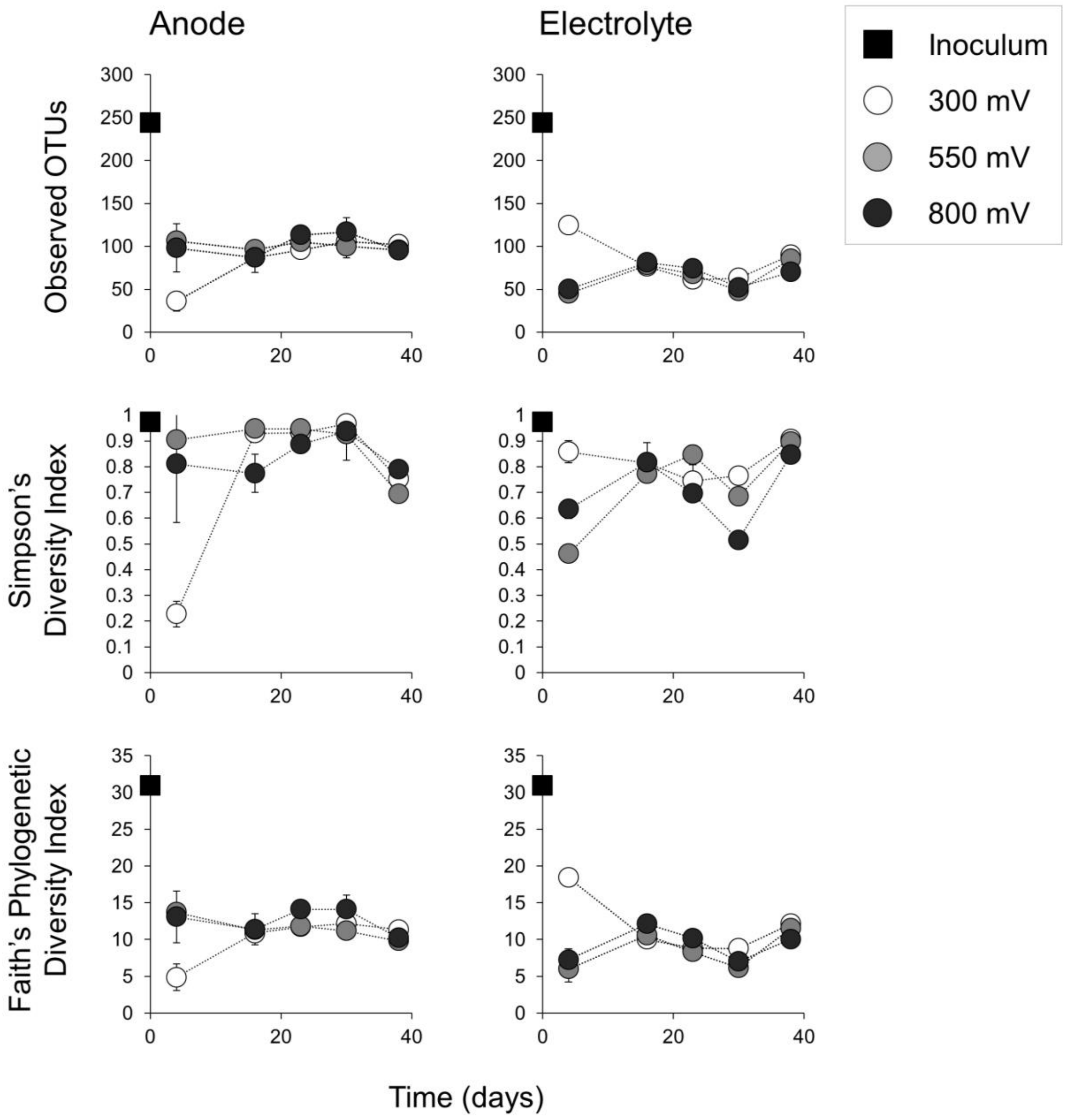

Fig. S4 Alpha diversity of microbial communities associated with the inoculum and the reactors over time.

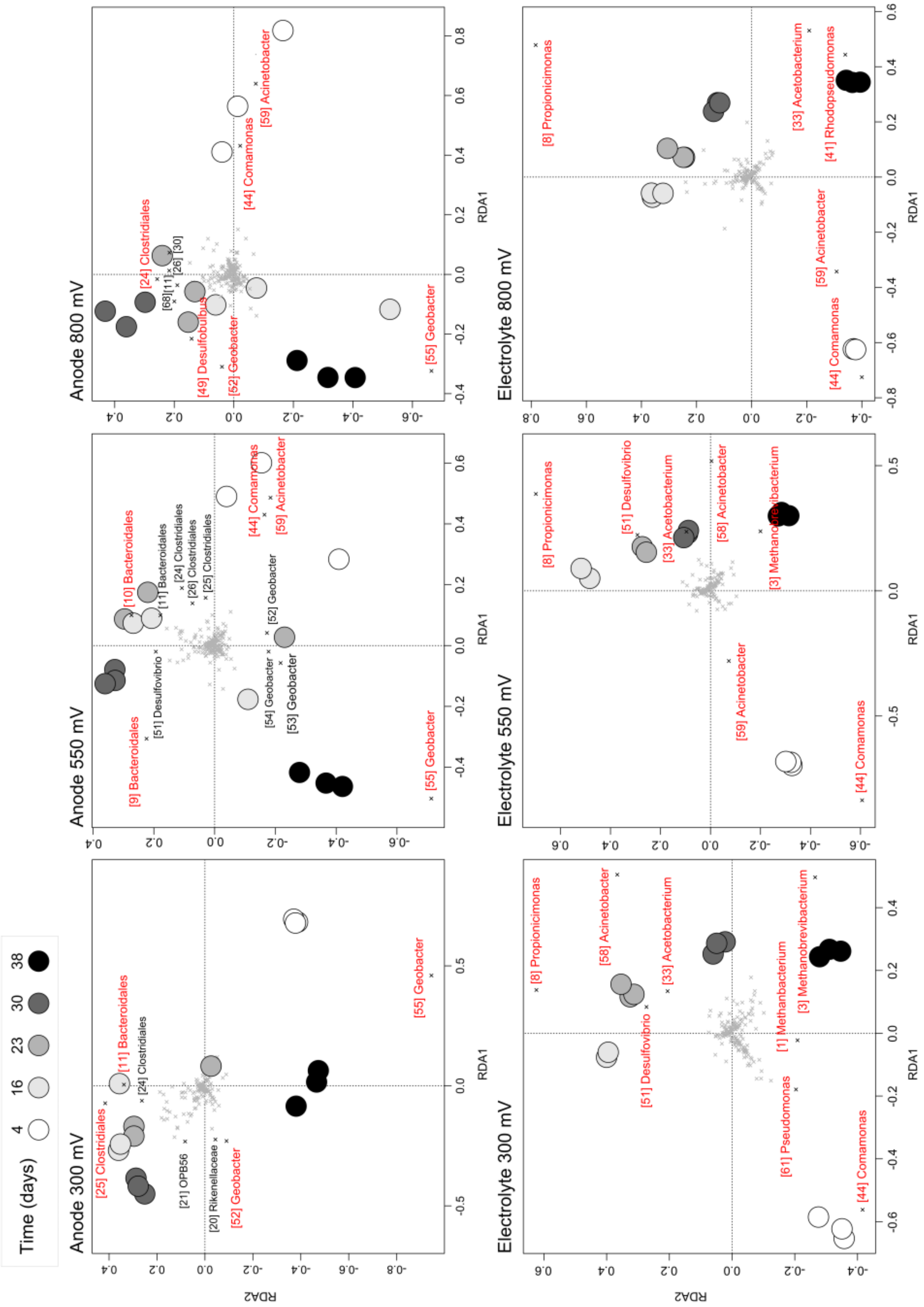

Fig. S5 Redundancy analysis ordinations summarising differences in the composition of microbial communities in the reactors over-time. OTUs are shown as crosses and those that discriminate between treatments are labelled consistently with the heatmap and other figures.

Table S1 Cumulative charge over time (mA h)

Day	300 mV	550 mV	800 mV
**4**	408±36	10±0.2	22±0.3
**16**	24,648±126	12,260±158	3,717±94
**23**	40,999±209	29,044±180	10,960±19
**31**	61,169±193	48,840±239	18,909±92
**39**	78,599±189	71,070±185	32,007±142

Supplementary methods

Feed solution for the anodes

The anode compartments were fed a mixture of volatile fatty acids (0.476 ml l^{-1} acetic acid, 0.231 ml l^{-1} propionic acid, 0.206 ml l^{-1} butyric acid, 0.093 ml l^{-1} iso-butyric acid, 0.096 ml l^{-1} valeric acid, 0.143 ml l^{-1} iso-valeric and 0.070 ml l^{-1} caproic acid; Freguia *et al.*, 2010), in modified M9 medium (0.5 g l^{-1} NaCl, 0.465 g l^{-1} $MgSO_4.H_2O$, 3 g l^{-1} Na_2HPO_4, 1.5 g l^{-1} KH_2PO_4, 0.5 g l^{-1} NH_4Cl, 14.7 mg l^{-1} $CaCl_2$, and 1 ml l^{-1} of a mixed trace element solution containing: 1 g l^{-1} $FeSO_4.7H_2O$, 70 mg l^{-1} $ZnCl_2$, 100 mg l^{-1} $MnCl_2.4H_2O$, 6 mg l^{-1} H_3BO_3, 130 mg l^{-1} $CaCl_2.6H_2O$, 2 mg l^{-1} $CuCl_2.2H_2O$, 24 mg l^{-1} $NiCl_2.6H_2O$, 36 mg l^{-1} $Na_2Mo_4.2H_2O$ and 238 mg l^{-1} $CoCl_2.6H_2O$).

VFA utilisation profiles

Volatile fatty acid (VFA) content was determined by injecting 0.9 ml sample mixed with 0.1 ml 10% formic acid into a GC fitted with a polar capillary column (DB-FFAP) at 140 °C and a flame ionization detector at 250 °C. Chemical oxygen demand (COD) measurements were performed according to the dichromate method (Greenberg et al., 1992)

PCR amplification and pyrosequencing

Universal 16S rRNA genes were amplified by PCR in 50 µl volumes containing 20 ng DNA, molecular biology grade water, 1X PCR Buffer minus Mg (Invitrogen), 50 nM of each of the dNTPs (Invitrogen), 1.5 mM $MgCl_2$ (Invitrogen), 0.3 mg BSA (New England Biolabs), 0.02 U *Taq* DNA Polymerase (Invitrogen), 8 µM each of the primers 926F and 1392R (Engelbrektson et al., 2010) modified on the 5' end to contain the 454 FLX Titanium Lib L adapters B and A, respectively. The reverse primers also contained a 5-6 base barcode sequence positioned between the primer sequence and the adapter. A unique bar-code was used for each sample. Thermocycling conditions were as follows: 95°C for 3 min; then 30 cycles of 95°C for 30 s, 55°C for 45 s, 72°C for 90 s; then 72°C for 10 min. Amplifications were performed using a Veriti® 96-well thermocycler (Applied Biosystems). Amplicons were purified using a QIAquick PCR purification kit (Qiagen), quantified using a Qubit™

fluorometer with a Quant-iT dsDNA BR Assay Kit and then normalised to 25 ng μl^{-1} and pooled for 454 pyrosequencing.

References

Engelbrektson A, Kunin V, Wrighton KC, Zvenigorodsky N, Chen F, Ochman H, et al. (2010). Experimental factors affecting PCR-based estimates of microbial species richness and evenness. ISME J. 4:642-647.

Freguia, S., Teh, E.H., Boon, N., Leung, K.M., Keller, J., Rabaey, K., 2010. Microbial fuel cells operating on mixed fatty acids. Bioresour. Technol. 101, 1233–1238.

Greenberg A, Clesceri LS, Eaton AD. 1992. Standard methods for the examination of water and wastewater, eighteenth ed. American public health association, Washington.
